# Progress of Diseases of the Throat and Nose

**Published:** 1902-02-01

**Authors:** 


### Progress of Diseases of the Throat and Nose.

(*Continued from page* 290.)

**Septal Deflections.**—Harrison Allen's supralabial operation is suited for cases where no overhang exists, and the extreme point of deflection extends above the anterior nasal spine, the anterior part of the vomer being then often deflected also. Ames Bliss [24] reminds us that Harrison Allen's operation consists in the division through, or immediately above, the maxillary crest, thus making the anterior part of the septum freely movable. The frænum of the upper lip having been divided, a small strong chisel is inserted through the wound and carefully pushed upward in the median line until the maxillary crest is reached. Then with a few light blows of the hammer it is driven horizontally backwards through the anterior nasal spine as far as the nasopalatine foramen. If the operator has been careful to direct the chisel so as to completely divide the maxillary crest, a finger introduced into the occluded nostril will at once force the septum over as far as desired. A rubber tube should then be placed in the occluded nostril. Any ridges existing along the line of attachment between the vomer and the triangular cartilage can be removed at the time of the septal replacement, or subsequently under cocaine. Ames Bliss has obtained uniformly satisfactory results in the large number of cases to which this operation is applicable, though it is almost unknown to the profession.

**Nasal Polypus.**—The removal by the curette of the diseased bony walls of the ethmoidal cells in nasal polypus and ethmoiditis is advocated by some rhinologists, but Beaman Douglas [25] records four cases of emphysema of the upper eyelid from nasal lesions, and two of these were due to operation on the ethmoidal cells. He comes to the following conclusions : (1) That the curette should be avoided as much as possible, for he believes that the curette especially causes traumatism of the lamina papyracea, and that alligator forceps or cutting forceps are better instruments. (2) He advises that no part of the middle turbinate bone should be amputated, as it forms a valuable guide along which operation may be carried out without risk of entering either the orbit or brain cavity. An ingenious method of passing a wire snare around the neck of a post-nasal polypus is described by Francis,[26] of Brisbane. His plan is as follows : pass a small, soft-rubber catheter, to the end of which a length of silk is attached, along the floor of the nostril on the affected side. Seize the end of the catheter as it emerges from behind the palate with a pair of forceps; cut the silk, and, retaining it, withdraw the catheter. Tie the mouth end of the silk to the end protruding from the nostril. Repeat the operation, only make sure that the catheter this time passes above the neck of the polypus. Release the end of the second silk, and withdraw the catheter. Then tie together the two mouth ends of the silks, and draw gently on the nasal ends. It is well to see that the loop thus made passes up well against the side of the pharynx to which the polypus is attached in the nose. We thus get a silken thread round the pedicle without having interfered with the polypus in any way. Next heat the last inch of a long piece of snare wire to take the temper out of the steel; make a small eyelet in the end and tie on one end of the silk. By gently pulling on the free end of the silk and easing the wire into the nose, the wire is passed round the pedicle. Thread the wire ends into a Jervis snare, and slowly wind off.

**Nasal Polypus and Spasmodic Asthma.**—Though reported some time back, the importance of two examples in men of severe and prolonged attacks of asthma associated with, and apparently dependent upon, the presence of nasal polypi, is worthy of special note. These cases were recorded by Walker Downie,[27] and he states that extirpation of the nasal polypi resulted in complete immunity from asthmatic symptoms. The first case was that of a male, aged 41, who for four months previous to the operation had been unable to attend to business, the attacks being almost continuous night and day. The left nasal passage was completely blocked by several large polypi; the right, too, was partially blocked. Immediately after the complete removal of the polypi the asthmatic attacks entirely disappeared. The second patient was also a male, aged 42, and the asthmatic attacks were likewise so severe as to necessitate his giving up all business. Polypi were found and removed from both nasal passages, together with the anterior halves of the middle turbinals. Immediate relief followed and was maintained.

**Diseases of the Maxillary Antrum.**—The commoner symptoms of antral empyema are now widely recognised, but in some cases the symptoms are most indefinite. Bronner [28] mentions a case of a medical man who suffered from recurrent unilateral tonsilitis and a bad smell in the nose ; examination revealed empyema of the antrum. The same authority has published three cases of antral empyema in which the only symptom was a bad smell from the nose, perceived by the patient. In cases of ozæna he finds the antrum is often affected, and it is obviously impossible to cure the ozæna until the antrum has been treated. The only way of making a reliable diagnosis of empyema is to puncture and to wash out the antrum; this is generally done with a Lichtwitz trocar. The opening is made in the lower meatus, about three centimetres behind the anterior part of the lower turbinated bone, and just under it. If made too low down the puncture is difficult and painful, as the bone is often very thick there. If cocaine or eucaine is applied a few minutes before operating, there is little or no pain. Warm sterilised water is injected through the trocar. It escapes through one of the ostia. If it is clear, of course there can be no empyema. If the ostia are blocked one must make a counter-opening or use a double trocar. *Cysts* are not very common, and they are mostly of dental origin. There are, roughly speaking, two kinds of dental cysts, those caused by retention of unerupted teeth and those due to inflammatory changes in the root-membrane of an already erupted tooth. In the first class the tooth is generally contained in the cyst. It is often difficult to diagnose between empyema and a cyst. A large cyst causes distension of the cheek, and on pressure you hear a peculiar crackling noise. If water is injected into the cyst, it does not pass out through the nares, as it would if injected into the antrum. *Tumours* of the antrum are frequently met with. The most common is carcinoma. The first symptoms are severe pain in the cheek and nasal discharge. One generally finds nasal polypi. These bleed freely on removal. If diagnosed early and if the whole of the upper jaw is removed (this is absolutely necessary) the prognosis is fairly good.

**Antrum of Highmore.**—A case of empyema of the antrum in a child, aged $2\frac{1}{2}$ years, is reported by Emil Mayer.[29] It followed scarlet fever, and later, severe nasal diphtheria. The pus contained streptococci and staphylococci, but no Klebs-Loeffler bacilli were found. The author states that in all the reported cases (there were six others only), the symptoms had been the same, viz., fistula under the eye, usually discharging pus, ectropion, one-sided purulent discharge from the nose, with foul odour and eroded bone.

**Mercurol.**—A compound of mercury with nucleinic acid (containing about 10 per cent. of mercury), if used in 5 per cent. solution, is a non-corrosive and non-irritant antiseptic solution which has the advantage of not precipitating albumin. Unfortunately the solution must be freshly made, and Lake finds that in order to obtain the most efficient action of mercurol, it should not be kept more than a week. He has found it specially suitable for irrigating cavities, such as the maxillary sines, owing to its non-irritant qualities.

[24] New York Med. Rec., July 13, 1901. [25] New York Med. Jour., March 23, 1901. [26] Australasian Medical Gazette, July 20, 1901. [27] Glasgow Med. Jour., October 1901. [28] Lancet, May 11, 1901. [29] New York Med. Rec., July 13, 1901.

